# A phase 1 study of oral ridaforolimus in pediatric patients with advanced solid tumors

**DOI:** 10.18632/oncotarget.12450

**Published:** 2016-10-04

**Authors:** Andrew D.J. Pearson, Sara M. Federico, Isabelle Aerts, Darren R. Hargrave, Steven G. DuBois, Robert Iannone, Ryan D. Geschwindt, Ruixue Wang, Frank G. Haluska, Tanya M. Trippett, Birgit Geoerger

**Affiliations:** ^1^ Paediatric Drug Development Unit, Children and Young People's Unit, Institute of Cancer Research, The Royal Marsden NHS Foundation Trust, Sutton, United Kingdom (Retired); ^2^ Department of Pediatric Oncology, St. Jude Children's Research Hospital, Memphis, TN, USA; ^3^ Department of Pediatric, Adolescent and Young Adult Oncology, Institut Curie, Paris, France; ^4^ Haematology and Oncology Department, Great Ormond Street Hospital for Children, London, United Kingdom; ^5^ Department of Pediatrics, University of California San Francisco School of Medicine, and Benioff Children's Hospital, San Francisco, CA, USA (current affiliation: Dana-Farber/Boston Children's Cancer and Blood Disorders Center and Harvard Medical School, Boston, MA, USA); ^6^ Clinical Research, Merck & Co., Inc., North Wales, PA, USA; ^7^ BARDS, MSD R&D (China) Co. Ltd., Beijing, China; ^8^ Clinical Research & Development, ARIAD Pharmaceuticals, Inc., Cambridge, MA, USA; ^9^ Department of Pediatrics, Memorial Sloan Kettering Cancer Center, New York, NY, USA; ^10^ Department of Childhood and Adolescent Oncology, Gustave Roussy, University Paris-Sud, Villejuif, France

**Keywords:** phase I-III trials_pediatric cancers, phase I-III trials_sarcoma/soft-tissue malignancies, ridaforolimus, mTOR, pharmacokinetics

## Abstract

**Purpose:**

Ridaforolimus is an investigational, potent, selective mTOR inhibitor. This study was conducted to determine the recommended phase 2 dose (RP2D), maximum tolerated dose, safety, pharmacokinetics, and antitumor activity of oral ridaforolimus in children with advanced solid tumors.

**Experimental Design:**

In this phase 1, multicenter, open-label study in children aged 6 to <18 years with advanced solid tumors, ridaforolimus was administered orally for 5 consecutive days/week in 28-day cycles until progression, unacceptable toxicity, or consent withdrawal. Dose started at 22 mg/m^2^ and increased to 28 mg/m^2^ and 33 mg/m^2^, followed by expansion at the RP2D.

**Results:**

Twenty patients were treated; 18 were evaluable for dose-limiting toxicities. One dose-limiting toxicity (grade 3 increased alanine aminotransferase) occurred in 1 patient at 33 mg/m^2^. Dose escalation concluded at 33 mg/m^2^; the maximum tolerated dose was not determined. The most common treatment-related adverse events (frequency ≥40%) were manageable grade 1–2 stomatitis, thrombocytopenia, hypertriglyceridemia, increased alanine aminotransferase, fatigue, hypercholesterolemia, anemia, and increased aspartate aminotransferase. Ridaforolimus exposure at 28 mg/m^2^ and 33 mg/m^2^ exceeded adult target levels. The RP2D for oral ridaforolimus in children was defined as 33 mg/m^2^. Four patients received at least 4 cycles; 2 with pineoblastoma and diffuse intrinsic pontine glioma had stable disease for 12 and 46 cycles, respectively.

**Conclusions:**

Ridaforolimus is orally bioavailable and well tolerated in children with advanced solid tumors. The RP2D (33 mg/m^2^, 5 days/week) exceeds the adult RP2D. The favorable toxicity and pharmacokinetic profiles may allow for combination therapy, a promising therapeutic option in pediatric malignancies.

## INTRODUCTION

The serine/threonine kinase mTOR is a central regulator of cell growth mediated by the phosphatidylinositol 3-kinase (PI3K)/AKT pathway [1-3]. Deregulation of the PI3K/AKT pathway is associated with tumorigenesis in a range of human cancers, including several pediatric malignancies [4-7], and inhibition of this pathway is a promising therapeutic strategy. When tested in the US National Cancer Institute–supported Pediatric Preclinical Testing Program, rapamycin, the prototypic mTOR inhibitor, induced significant differences in event-free survival distributions compared with controls in 27 of 36 in vivo solid tumor models, and objective responses were observed in select osteosarcoma, rhabdomyosarcoma, and rhabdoid tumor xenografts [8]. Inhibition of mTOR decreases VEGF expression in human rhabdomyosarcoma cell lines and xenografts, reduces tumor angiogenesis in human rhabdomyosarcoma xenografts [9, 10], and decreases VEGF expression and ezrin-mediated metastatic and invasive behavior in murine osteosarcoma cell lines [11, 12], suggesting that mTOR inhibition may be promising for treatment of sarcomas. Prolonged stable disease was observed in a phase 2 study of single-agent therapy with the mTOR inhibitor temsirolimus in pediatric patients with neuroblastoma and high-grade glioma [13]. Results from a Children's Oncology Group trial in rhabdomyosarcoma demonstrated superior event-free survival in patients randomized to receive treatment with temsirolimus in combination with vinorelbine and cyclophosphamide versus treatment with bevacizumab, vinorelbine, and cyclophosphamide [14]. This growing body of preclinical and clinical evidence has increased interest in mTOR inhibitor therapies for pediatric solid tumors.

Ridaforolimus (formerly deforolimus, AP23573, or MK-8669) is an orally bioavailable non-prodrug analog of rapamycin [15] in clinical development for a variety of solid tumors. Studies have shown that ridaforolimus selectively and potently inhibits mTOR function and proliferative activity in different human tumor cell lines in vitro and tumor xenograft models in vivo, and has synergistic activity when combined with other anticancer agents, such as doxorubicin and carboplatin/paclitaxel [16, 17]. In phase 1 and 2 trials, ridaforolimus was well tolerated and demonstrated clinical activity in adults with advanced solid tumors, including those with various sarcomas [18-22]. The phase 3 SUCCEED (Sarcoma Multicenter Clinical Evaluation of the Efficacy of Ridaforolimus) trial reported that oral ridaforolimus reduced the risk of progression or death by 28% compared with placebo in patients with advanced soft tissue and bone sarcomas who had benefited from the immediately preceding cytotoxic chemotherapy [23]. Preliminary data suggest that ridaforolimus is well tolerated and has clinical activity in pediatric patients. In the SUCCEED trial, 7 pediatric patients aged 13 to 17 years received ridaforolimus; 1 had a partial response, 4 had stable disease, and 2 had progressive disease [24]. Results from a phase 1 study in pediatric patients aged 2 to 16 years showed that intravenous ridaforolimus (8-16 mg/m^2^ daily for 5 consecutive days every other week) was well tolerated and associated with stable disease in 6 (40%) of 15 evaluable pediatric patients with heavily pretreated solid tumors, 4 of 6 with central nervous system tumors and 2 of 8 with sarcomas [25].

The primary objectives of this phase 1 study (NCT01431534) were to define the dose-limiting toxicities (DLTs), maximum tolerated dose (MTD), and recommended phase 2 dose (RP2D), and to characterize the pharmacokinetics of ridaforolimus when orally administered to children and adolescents aged 6 to <18 years with advanced solid tumors. Antitumor activity was assessed as a secondary objective. Results from this study informed ridaforolimus dose selection for the companion phase 1 study (NCT01431547), which investigated dalotuzumab monotherapy and ridaforolimus-dalotuzumab combination therapy in pediatric patients with advanced solid tumors [26].

## RESULTS

### Patients

**Figure 1:**
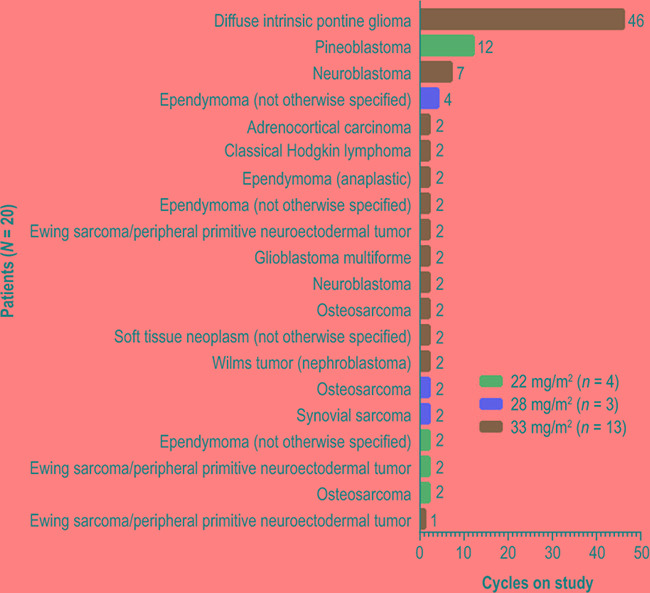
Time on study Each bar shows the number of 28-day cycles that a patient received the study treatment, oral ridaforolimus (22, 28, or 33 mg/m^2^) administered once daily for 5 days per week.

Twenty patients were enrolled and treated at ridaforolimus dose levels of 22 mg/m^2^ (*n* = 4), 28 mg/m^2^ (*n* = 3), and 33 mg/m^2^ (*n* = 13). Median age was 13 years (range, 8–17 years) and 60% of patients were female (Table 1). Tumor diagnoses included: ependymoma in 4 patients (1 anaplastic and 3 not otherwise specified); Ewing sarcoma/peripheral primitive neuroectodermal tumor and osteosarcoma in 3 patients each; neuroblastoma in 2 patients; and other diagnoses in 8 patients. Approximately half of the patients had received at least 2 prior anticancer regimens (Table 1). Patients received between 1 and 46 cycles of study treatment (Figure 1). The mean number (± SD) of cycles based on ridaforolimus dose levels of 22, 28, and 33 mg/m^2^ was 4.5 ± 5.0, 2.7 ± 1.2, and 5.7 ± 12.2 cycles, respectively; medians and ranges are shown in Table 2. All patients in the 22 mg/m^2^ (*n* = 4) and 28 mg/m^2^ (*n* = 3) groups discontinued the study due to progressive disease. In the 33 mg/m^2^ (*n* = 13) group, 10 patients (77%) discontinued due to progressive disease, 1 (8%) discontinued due to an adverse event (grade 5 gastric perforation related to underlying disease), and 2 patients (15%; 1 with pineoblastoma and 1 with diffuse intrinsic pontine glioma) entered the extension study, in which treatment was continued. Four patients received at least 4 courses, 1 with ependymoma (4 cycles at 28 mg/m^2^), 1 with neuroblastoma (7 cycles at 33 mg/m^2^), 1 with pineoblastoma (12 cycles at 22 mg/m^2^), and 1 with diffuse intrinsic pontine glioma (46 cycles at 33 mg/m^2^ as of January 2016).

**Table 1:** Patient characteristics

	Total *N* = 20	Ridaforolimus 22 mg/m^2^ *n* = 4	Ridaforolimus 28 mg/m^2^ *n* = 3	Ridaforolimus 33 mg/m^2^ *n* = 13
Median age, years (range)	13.0 (8–17)	13.5 (8–17)	15.0 (12–17)	12.0 (8–17)
Gender, *n* (%)				
Male	8 (40)	1 (25)	2 (67)	5 (38)
Female	12 (60)	3 (75)	1 (33)	8 (62)
Tumor diagnosis, *n* (%)				
Ependymoma (1 anaplastic and 3 NOS)	4 (20)	1 (25)	1 (33)	2 (15)
Ewing sarcoma/peripheral primitive neuroectodermal tumor	3 (15)	1 (25)	0	2 (15)
Osteosarcoma	3 (15)	1 (25)	1 (33)	1 (8)
Neuroblastoma	2 (10)	0	0	2 (15)
Other^a^	8 (40)	1 (25)	1 (33)	6 (46)
Number of prior therapies, *n* (%)				
1	8 (40)	2 (50)	1 (33)	5 (38)
2	3 (15)	0	0	3 (23)
3	3 (15)	1 (25)	0	2 (15)
≥4	4 (20)	1 (25)	2 (67)	1 (8)
Unknown	2 (10)	0	0	2 (15)

a *Other* includes 1 patient each with: adrenocortical carcinoma; classical Hodgkin lymphoma; diffuse intrinsic pontine glioma; glioblastoma multiforme; pineoblastoma; soft tissue neoplasm, NOS; synovial sarcoma; and Wilms tumor (nephroblastoma).

NOS, not otherwise specified.

**Table 2:** Dose-escalation and assessment of DLTs in pediatric patients treated with oral ridaforolimus (*N* = 20)

Dose level	Dose, mg/m^2^	Patients treated, *n*	Patients evaluable for DLT, *n*	Patients with DLT, *n*	Time to DLT onset, days	Median (range) time on therapy, cycles
1	22	4	3	0	—	2 (2–12)
2	28	3	3	0	—	2 (2–4)
3	33	7	6	1 (grade 3 increased ALT)	22 (cycle 1)	2 (1–46)^a^
RP2D expansion	33	6	6	0	—	—

a Cycles on therapy for all patients treated with ridaforolimus 33 mg/m^2^ during dose escalation and RP2D expansion (*n* = 13).

### Dose-limiting toxicity and RP2D

A total of 18 patients were evaluable for DLTs (Table 2). Two patients were not evaluable for DLTs and were replaced for the DLT analysis only: 1 patient with concomitant administration of a high-dose corticosteroid, which could have affected the determination of DLTs, and 1 patient who had progressive disease before completing the DLT assessment period. One DLT of grade 3 increased alanine aminotransferase (ALT) occurred in 1 patient at the 33 mg/m^2^ dose. Drug administration was interrupted and the DLT resolved. Dose escalation was stopped at the highest planned dose level explored in this study, 33 mg/m^2^, and the MTD was not determined. Based on the toxicity profile, pharmacokinetics, and efficacy (all reported below), the RP2D for oral ridaforolimus in children was defined as 33 mg/m^2^, and the expansion phase was at this dose.

### Adverse events

Nineteen patients (95%) experienced at least 1 treatment-related adverse event. Table 3 shows treatment-related adverse events reported in more than 1 patient. Treatment-related adverse events reported in ≥30% of patients were stomatitis (75%), thrombocytopenia (65%), hypertriglyceridemia (50%), increased ALT (50%), fatigue (45%), hypercholesterolemia (45%), anemia (40%), increased aspartate aminotransferase (AST) (40%), leukopenia (35%), nausea (30%), and neutropenia (30%).

**Table 3:** Treatment-related adverse events overall and in more than 1 pediatric patient treated with ridaforolimus

	Total *N* = 20		Ridaforolimus 22 mg/m^2^ *n* = 4		Ridaforolimus 28 mg/m^2^ *n* = 3		Ridaforolimus 33 mg/m^2^ *n* = 13	
	Any grade	Grades 3–4	Any grade	Grades 3–4	Any grade	Grades 3–4	Any grade	Grades 3–4
Patients with ≥1 treatment-related adverse event	19 (95)	9 (45)	3 (75)	2 (50)	3 (100)	2 (67)	13 (100)	5 (38)
Nonhematologic								
Stomatitis	15 (75)	0	2 (50)	0	3 (100)	0	10 (77)	0
Fatigue	9 (45)	0	2 (50)	0	1 (33)	0	6 (46)	0
Nausea	6 (30)	0	1 (25)	0	1 (33)	0	4 (31)	0
Dysgeusia	3 (15)	0	0	0	0	0	3 (23)	0
Headache	3 (15)	0	0	0	0	0	3 (23)	0
Photophobia	3 (15)	0	0	0	1 (33)	0	2 (15)	0
Vomiting	3 (15)	0	1 (25)	0	0	0	2 (15)	0
Decreased appetite	2 (10)	0	0	0	0	0	2 (15)	0
Decreased weight	2 (10)	0	0	0	0	0	2 (15)	0
Diarrhea	2 (10)	0	0	0	0	0	2 (15)	0
Dry mouth	2 (10)	0	2 (50)	0	0	0	0	0
Epistaxis	2 (10)	0	0	0	0	0	2 (15)	0
Oral herpes	2 (10)	1 (5)	1 (25)	1 (25)	0	0	1 (8)	0
Rash	2 (10)	0	0	0	1 (33)	0	1 (8)	0
Hematologic								
Thrombocytopenia^a^	13 (65)	2 (10)	2 (50)	0	1 (33)	1 (33)	10 (77)	1 (8)
Anemia^a^	8 (40)	0	2 (50)	0	1 (33)	0	5 (38)	0
Leukopenia^b^	7 (35)	0	1 (25)	0	2 (67)	0	4 (31)	0
Neutropenia^a^	6 (30)	1 (5)	2 (50)	0	0	0	4 (31)	1 (8)
Lymphopenia^a^	2 (10)	1 (5)	1 (25)	0	0	0	1 (8)	1 (8)
Biochemical								
Hypertriglyceridemia^a^	10 (50)	1 (5)	2 (50)	0	1 (33)	0	7 (54)	1 (8)
Increased ALT	10 (50)	5 (25)	1 (25)	1 (25)	1 (33)	1 (33)	8 (62)	3 (23)
Hypercholesterolemia^a^	9 (45)	1 (5)	2 (50)	0	1 (33)	1 (33)	6 (46)	0
Increased AST	8 (40)	1 (5)	1 (25)	0	1 (33)	0	6 (46)	1 (8)
Hypophosphatemia^a^	5 (25)	1 (5)	2 (50)	1 (25)	1 (33)	0	2 (15)	0
Increased γ-glutamyltransferase	5 (25)	1 (5)	1 (25)	0	1 (33)	0	3 (23)	1 (8)
Hyperglycemia^a^	2 (10)	0	0	0	1 (33)	0	1 (8)	0
Hypocalcemia^a^	2 (10)	0	1 (25)	0	0	0	1 (8)	0
Hypokalemia^a^	2 (10)	0	1 (25)	0	1 (33)	0	0	0
Increased alkaline phosphatase	2 (10)	0	1 (25)	0	0	0	1 (8)	0
Increased bilirubin	2 (10)	0	1 (25)	0	0	0	1 (8)	0

Numbers are *n* (%).

*Note:* Only the highest reported grade of a given adverse event is counted for an individual patient. Three grade 5 adverse events occurred during the study (neurologic symptoms in a patient with central nervous system metastases, neoplasm progression, and gastric perforation related to underlying disease); none were related to ridaforolimus, according to the investigator. The number of cycles evaluated is shown in Table 2.

a Reported as an adverse event by the investigator or based on laboratory values.

b Based on laboratory values only; leukopenia was not reported separately by investigators as a treatment-related adverse event.

Treatment-related grade 3/4 adverse events were reported in 9 patients (45%). The most common treatment-related grade 3/4 adverse events were increased ALT (25%) and thrombocytopenia (10%). Serious adverse events occurred in 12 patients (60%), most of which were disease related. Three patients (15%) experienced serious adverse events related to study treatment: 1 patient at 22 mg/m^2^ (grade 3 oral herpes) and 2 patients at 33 mg/m^2^ (grade 2 rectal pain in 1 patient and grade 2 seizure in 1 patient). Three deaths occurred during the study, 1 due to neurologic symptoms in a patient with central nervous system metastases, 1 due to neoplasm progression, and 1 due to gastric perforation related to underlying disease. No deaths were considered related to ridaforolimus.

### Pharmacokinetics

**Figure 2:**
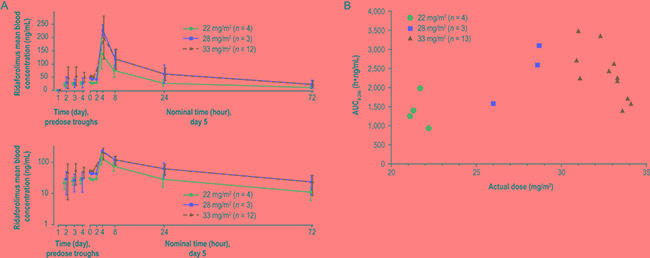
Pharmacokinetic profile of oral ridaforolimus in pediatric patients **A.** Mean (± SD) blood concentration of ridaforolimus following oral dosing at 22, 28, and 33 mg/m^2^ once daily for 5 days per week of a 28-day cycle in pediatric patients. Top panel: linear scale; bottom panel: semi-log scale. **B.** Exposure (AUC_0–24h_) relative to actual dose of oral ridaforolimus (calculated based on body surface area) in pediatric patients.

Blood samples for pharmacokinetic evaluation were collected from all treated patients, and pharmacokinetic data were evaluable for 19 patients. One patient was excluded because no dose was administered on day 5. Mean blood concentrations of ridaforolimus following oral dosing are shown by dose level in Figure 2A, and exposure relative to actual dose is plotted in Figure 2B. Trough concentrations from predose samples on days 1 through 5 indicated that steady-state concentrations had been achieved at day 5. Absorption occurred after a lag of approximately 2 hours after dosing, and highest blood concentrations were generally observed 4 hours after dosing. Concentration-versus-time profiles were lowest at the 22 mg/m^2^ dose, but nearly superimposable at the 28 mg/m^2^ and 33 mg/m^2^ doses. Exposure at the 28 mg/m^2^ and 33 mg/m^2^ dose levels (day 5 area under the concentration-versus-time curve from 0 to 24 hours [AUC_0–24h_] = 2,330 and 2,280 h•ng/mL, respectively) was approximately 34% higher and 31% higher, respectively, than target levels based on the adult RP2D of 40 mg (once daily for 5 days per week) in adults with a mean body surface area (BSA) of 1.8 m^2^ (day 5 AUC_0__–24h_ = 1,739 h•ng/mL; data on file with ARIAD Pharmaceuticals, Inc./Merck & Co., Inc.). Pharmacokinetic parameters are summarized in Table 4. Mean (geometric) elimination half-lives were 26 to 27 hours, independent of dose level. None of the pharmacokinetic parameters appeared to vary with patient age.

**Table 4:** Pharmacokinetic parameters for blood concentrations of ridaforolimus on day 5 of oral dosing (once daily × 5 days per week) according to dose level in pediatric patients

Pharmacokinetic parameter	Dose level 1 22 mg/m^2^ (*n* = 4)	Dose level 2 28 mg/m^2^ (*n* = 3)	Dose level 3 33 mg/m^2^ (*n* = 12)^a^
Day 5 AUC_0–24h_, h•ng/mL	*n* = 4	*n* = 3	*n* = 11
Geometric mean (CV%^b^)	1,340 (32)	2,330 (36)	2,280 (30)
C_4h_, ng/mL	*n* = 4	*n* = 3	*n* = 11
Arithmetic mean (SD)	139 (57)	228 (21)	200 (81)
Geometric mean (CV%^b^)	131 (41)	227 (10)	184 (45)
C_8h_, ng/mL	*n* = 4	*n* = 3	*n* = 12
Arithmetic mean (SD)	74 (23)	119 (35)	118 (39)
Geometric mean (CV%^b^)	71 (37)	116 (32)	113 (32)
t_1/2_, h	*n* = 3^c^	*n* = 3	*n* = 9
Arithmetic mean (SD)	25.5 (2.8)	28.2 (9.5)	26.9 (8.7)
Median (range)	24.5 (23.5–28.7)	26.5 (19.7–38.4)	24.9 (21.0–49.1)
Geometric mean (CV%^b^)	25.5 (10.5)	27.2 (34.5)	26.0 (26.7)

a Thirteen patients were enrolled with daily dose administration of ridaforolimus 33 mg/m^2^; 1 patient was excluded because of no exposure on day 5. For 1 patient, AUC, C_4h_, and t_1/2_ were not evaluable because of limited sampling. For 2 additional patients, t_1/2_ was not evaluable. The geometric mean of BSADN AUC (*n* = 11) was 69.8 h•ng•m^2^/(mL•mg). The lower bounds of the 90% 1-sided confidence intervals for the geometric means of day 5 AUC_0–24h_, C_4h_, C_8h_, BSADN AUC, and t_1/2_ were 2,020 h•ng/mL, 154 ng/mL, 100 ng/mL, 61.2 h•ng•m^2^/(mL•mg), and 23.0 h, respectively. Backtransformed least-squares mean and confidence interval were performed on natural log-transformed values.

b Geometric coefficient of variation, where CV% = 100 × √(exp [S^2^] − 1) and S^2^ is the observed variance on the natural logarithmic scale.

c For 1 patient, t_1/2_ was not evaluable.

BSADN AUC, area under the concentration-versus-time curve normalized by BSA-adjusted dose; C_4h_, concentration at 4 hours; C_8h_, concentration at 8 hours; CV%, coefficient of variation as a percentage; t_1/2_, elimination half-life.

### Efficacy

None of the patients had a partial or complete response. However, 4 patients received at least 4 courses. One patient with pineoblastoma had stable disease for 12 cycles at the 22 mg/m^2^ dose level before disease progression (progression-free survival was 12.8 months). One patient with diffuse intrinsic pontine glioma treated at the 33 mg/m^2^ dose level continued to receive ridaforolimus with stable disease for 46 cycles as of January 2016.

## DISCUSSION

This is the first trial to evaluate the oral formulation of the mTOR inhibitor ridaforolimus in pediatric patients. In this phase 1 study, ridaforolimus was well tolerated in pediatric patients with advanced malignancies, with only 1 DLT (grade 3 increased ALT) at the highest dose of 33 mg/m^2^ once daily for 5 days per week. Stomatitis and thrombocytopenia were the main treatment-related toxicities and were mostly mild or moderate in severity. Dose escalation was stopped at 33 mg/m^2^, equivalent to 150% of the adult RP2D of 40 mg (assuming an average adult BSA of 1.8 m^2^) [27], and the MTD was not determined. The RP2D for oral ridaforolimus in children was defined as 33 mg/m^2^, although pharmacokinetic data indicate that 28 mg/m^2^ also achieves target concentrations and may also be considered for combination treatment.

The toxicity profile for oral ridaforolimus in children in this study is comparable to that observed with intravenous ridaforolimus in previous studies in adults [20, 27] and children [25]. In a phase 1 study in pediatric patients with refractory solid tumors, no DLTs were observed with intravenous ridaforolimus (8–16 mg/m^2^ daily for 5 consecutive days every other week) [25]. The most common adverse events in the intravenous pediatric study, mostly mild to moderate, were thrombocytopenia, anemia, hypertriglyceridemia, leukopenia, elevated AST and ALT, and hypophosphatemia [25]. In adults, DLTs of grade 2 stomatitis and oral mucositis were observed with oral ridaforolimus (the MTD was 40 mg for the once daily × 5 days per week regimen) and intravenous ridaforolimus (MTD, 18.75 mg/d) [20, 27]. The most common treatment-related adverse events in adults were grade 1 to 2 mucositis, rash, and anemia with intravenous administration [20] and fatigue and mucosal inflammation with oral administration [27]. Although the pediatric MTD of oral and intravenous ridaforolimus has not been identified, evidence of clinical benefit was noted in 2 (10%) of the 20 treated pediatric patients in our study and in 6 (40%) of 15 patients treated with intravenous ridaforolimus, 8 to 16 mg/m^2^ daily for 5 consecutive days every other week, in a previous pediatric study [25], suggesting that further dose escalation may not be necessary.

Although head-to-head trials have yet to be conducted, the safety profile of oral ridaforolimus may be considered in the context of other mTOR inhibitors evaluated in children with refractory solid tumors. In a phase 1 trial of temsirolimus administered once weekly to children with recurrent or refractory solid tumors, 1 DLT (grade 3 anorexia) was observed in 1 of 7 patients who received the highest dose tested (150 mg/m^2^) [28] and the phase 2 trial was performed at 75 mg/m^2^, which, as in the ridaforolimus study, was higher than the equivalent adult RP2D but was well tolerated [13]. A phase 1 trial of oral everolimus in children with refractory solid tumors reported DLTs of grade 3 increased ALT (*n* = 1), grade 3 mucositis (*n* = 1), and grade 3 diarrhea (*n* = 1) in 3 of 3 patients treated at the highest dose of 6.5 mg/m^2^ [29], and the RP2D of 5 mg/m^2^ is roughly equivalent to the recommended adult dose of 10 mg once daily [29, 30]. Ridaforolimus compares favorably in this study, with 1 DLT reported in 1 patient.

Although limited by the relatively small number of sampling time points, the pharmacokinetic profile of ridaforolimus in whole blood following oral administration to pediatric patients was comparable to that observed in previous studies of oral ridaforolimus in adult patients [21, 27]. Exposure with the 33 mg/m^2^ dose in children was approximately 31% higher than with the RP2D in adults (40 mg once daily, 5 days per week), suggesting that clinically effective concentrations are attained with the 33 mg/m^2^ dose. The concentration-versus-time profiles for the 28 mg/m^2^ and 33 mg/m^2^ doses were nearly superimposable, consistent with previous reports of a nonlinear relationship between dose and ridaforolimus concentration in whole blood [25, 27]. The reason the MTD of ridaforolimus was not reached may have been because higher doses do not produce higher exposures. Rapamycin analogs reach a plateau at higher doses due to saturable binding to intracellular protein FK506-binding protein in red blood cells [25]. The pharmacokinetic plateau observed in whole blood may not be observed in serum, because a higher unbound fraction is expected at higher concentrations due to saturation of FK506-binding protein in the red blood cells. This phenomenon supports the use of 33 mg/m^2^ rather than 28 mg/m^2^ as the RP2D. Although the dose could potentially be escalated further because the MTD was not determined, 33 mg/m^2^ appears to provide an appropriate exposure level in children, given that future development of ridaforolimus may involve combination therapy to increase efficacy.

Prolonged stable disease was observed in 2 patients in this study, 1 patient with pineoblastoma and 1 with diffuse intrinsic pontine glioma, with progression-free survival of 12.8 months and >42 months, respectively. This is consistent with observations that ridaforolimus crosses the blood–brain barrier [31] and that the benefit of single-agent therapy with an mTOR inhibitor is characteristically disease stabilization [25, 28, 29], including in patients with diffuse intrinsic pontine glioma [13]. While mTOR inhibitors have generally been associated with modest clinical activity as single-agent therapies, chemotherapy or other targeted agents have shown improved effects and even reversed resistance when combined with an mTOR inhibitor [14, 32, 33]. Preclinical studies of mTOR inhibitors combined with insulin-like growth factor 1 receptor (IGF1R) inhibitors showed additive or synergistic antitumor activity when the agents were combined [34-36], guiding interest in combining ridaforolimus with an anti-IGF1R monoclonal antibody, dalotuzumab. Two phase 1 studies [34, 37] have evaluated ridaforolimus in combination with dalotuzumab in adults and have shown that combining these agents is feasible and that the combination may have synergistic activity in advanced malignancies. A positive effect was not observed in a phase 2 study of ridaforolimus, dalotuzumab, and exemestane versus ridaforolimus and exemestane alone in advanced breast cancer [38]; however, lower doses of ridaforolimus in the first arm may have contributed to this lack of effect. In a phase 1 study of dalotuzumab monotherapy and ridaforolimus-dalotuzumab combination therapy in pediatric patients (NCT01431547), 1 of 20 patients who received dalotuzumab alone achieved a confirmed partial response, and none of the 4 patients who received the ridaforolimus-dalotuzumab combination achieved a response or prolonged stable disease [26]; however, it could be valuable to assess the ridaforolimus-dalotuzumab combination in a larger cohort. Ridaforolimus could be combined with other compounds, as well.

Validated biomarkers are not currently available for the prediction of efficacy and the selection of patients for future development of ridaforolimus as an mTOR inhibitor in pediatric oncology. Preclinical models and early-phase clinical trials have suggested that mutations in the *PIK3CA* gene, *PTEN* loss of function mutations, and high levels of phosphorylated AKT and phosphorylated S6 ribosomal protein may predict the efficacy of mTOR inhibitors [39, 40]. Future clinical trials should incorporate the investigation of biomarkers that may be predictive of response to treatment.

In conclusion, ridaforolimus is an investigational, orally bioavailable mTOR inhibitor that is well tolerated in pediatric patients with advanced solid tumor malignancies, with grade 3 increased ALT as a DLT. The safety profile observed in children is comparable to that observed in adults. The RP2D for ridaforolimus in children is 33 mg/m^2^, 5 days per week, although preliminary pharmacokinetic data suggest that 28 mg/m^2^ may also be considered. In view of the potency, oral bioavailability, and toxicity profile of ridaforolimus in the current trial, along with similar pharmacokinetic profiles observed with intravenous and oral ridaforolimus in previous studies in children and adults, oral ridaforolimus may be well suited for combination therapy, which may represent an important option for use in pediatric malignancies.

## MATERIALS AND METHODS

### Patients

Eligible patients were children ≥6 (due to tablet size) to <18 years of age at the date of enrollment with histologically or cytologically confirmed malignant solid tumors, including tumors of the central nervous system and lymphoma, that had progressed despite standard therapy or for which no effective standard therapy was known. Patients who had received standard therapy and continued to have biopsy-proven residual stable disease were also eligible. In the absence of a biopsy, patients with sarcoma were eligible on the basis of persistent positron emission tomography activity and patients with neuroblastoma were eligible on the basis of persistent activity by ^123^I-metaiodobenzylguanidine scan. Diagnoses of diffuse intrinsic pontine glioma did not require biopsy. Patients with measurable (per Response Evaluation Criteria in Solid Tumors, version 1.1 [RECIST v1.1]) or nonmeasurable disease were eligible. Patients were required to have a BSA within the acceptable range for a given dose cohort in order to achieve an actual dosing variation of ≤2 mg/m^2^ above or below the target (see dosing nomogram in Supplementary Table S1). Other key inclusion criteria were: Lansky Play Scale score of ≥70 for children <10 years of age; Karnofsky performance score of ≥70 for children aged 10 to 15 years; Eastern Cooperative Oncology Group performance status of 0 to 2 for patients ≥16 years of age; absolute neutrophil count of ≥1,000/μL, platelet count ≥75,000/μL, serum creatinine level ≤1.5 times the upper limit of normal (≤1.5 × ULN) for age, serum total bilirubin level ≤1.5 × ULN (or direct bilirubin at or below the ULN for patients with total bilirubin >1.5 × ULN), AST and ALT levels ≤2.5 × ULN (≤5 × ULN in patients with liver metastases), fasting serum glucose level <160 mg/dL, serum cholesterol level <350 mg/dL, and fasting triglyceride level <400 mg/dL. Key exclusion criteria were as follows: uncontrolled intercurrent illness; poorly controlled type 1 or 2 diabetes (fasting glucose level >160 mg/dL); known human immunodeficiency virus, hepatitis B virus, or hepatitis C virus infection (testing not required); or persistent acute toxicity (grade ≥2) from previous therapy, excluding alopecia, neuropathy, and hearing loss. Patients who previously received rapamycin analogs or were currently receiving any other investigational agents were excluded.

The study was conducted in accordance with the Declaration of Helsinki, Good Clinical Practice guidelines, and all local and federal regulations. The study protocol was approved by the Institutional Review Board or Ethics Review Committee at each participating site. Each patient's parent or legal guardian provided written informed consent, and patients provided assent according to local regulations.

### Study design and dose escalation

This phase 1, multicenter, open-label study was conducted at a total of 8 study sites (3 study sites in the United States and 5 Innovative Therapies for Children with Cancer [ITCC] Consortium centers [2 in the United Kingdom and 3 in France]) between January 31, 2012, and the data cutoff, January 14, 2014 (ClinicalTrials.gov identifier NCT01431534; European Clinical Trials Database [EudraCT] number 2011-000729-55). An enteric-coated tablet formulation of ridaforolimus was orally administered for 5 consecutive days and not administered for 2 consecutive days per week in 28-day cycles until progression, unacceptable toxicity, or consent withdrawal. Patients who did not have disease progression, who adequately tolerated therapy, and who continued to meet eligibility criteria for 6 months after completion of the enrollment period were eligible to enter an extension phase of the study. Dose escalation was conducted by using a modified toxicity probability intervals method [41] that targeted a 30% DLT rate and started at 22 mg/m^2^ (the equivalent of the adult RP2D [27], a 40-mg dose in adults with a BSA of 1.8 m^2^). The dose was escalated to 28 mg/m^2^ and 33 mg/m^2^ (increments of 25% of starting dose). Dosing was adjusted based on BSA and rounded to the nearest 10-mg tablet. Patients were to be treated at sequentially rising dose levels until a preliminary MTD was identified.

### Safety assessments

Toxicity was graded according to the National Cancer Institute Common Terminology Criteria for Adverse Events, version 4.0 [42]. Safety data were reported for all patients who received at least 1 dose of the study drug. A DLT was defined as any of the following events considered at least possibly related to the study drug and occurring during the first 28-day treatment cycle with adequate drug exposure (having received >75% of planned doses, exclusive of study drug doses missed due to treatment-related toxicity): grade 3 to 4 neutropenia associated with fever, antibiotics, or hospitalization for infection; grade 4 thrombocytopenia requiring platelet transfusion; grade 4 neutropenia, grade 4 thrombocytopenia, or grade ≥3 hyperglycemia persisting for ≥5 days; grade ≥3 diarrhea persisting >24 hours; grade ≥3 nausea, vomiting, or other nonhematologic toxicity persisting despite optimal medical management (except transient electrolyte abnormalities, transient grade 3 liver function test result elevations, grade 3 neurotoxicity in patients who had grade 3 neurotoxicity at baseline, and hearing loss in patients who had grade 3 hearing loss at baseline); or any toxicity considered at least possibly related to the study drug that prevents completion of the DLT assessment period, or interrupts dosing (or delays the next cycle) for >10 dosing days. Once the RP2D of ridaforolimus was defined, an expansion cohort to be treated at the RP2D was planned to obtain additional safety, tolerability, and pharmacokinetic data.

### Pharmacokinetic assessments

Whole blood samples (2 mL each) for pharmacokinetic analyses were collected at the following time points: <5 minutes before dosing on days 1 through 5 and at 0.5, 1, 2, 4, 8, 24, and 72 hours after drug administration on day 5 of the first week of the first treatment cycle. Blood concentrations of ridaforolimus were determined using a validated liquid chromatography/tandem mass spectrometry assay as in previous ridaforolimus studies (Charles River Laboratories, Shrewsbury, MA) [25, 27]. The pharmacokinetic parameters of AUC_0__–24h_ and elimination half-life (t_1/2_) were determined by noncompartmental modeling using WinNonlin software (Pharsight, Certara, Princeton, NJ).

### Efficacy assessments

All patients were to have computed tomography or magnetic resonance imaging scans at baseline, every 2 cycles during treatment, and at the time of treatment discontinuation. Local investigator assessment of treatment response was performed using RECIST v1.1 [43].

### Statistical analyses

Up to approximately 18 patients were to be treated during dose escalation depending on empirical safety data, with up to approximately 12 patients treated at the MTD (or maximum dose if the MTD was not achieved). Assuming that the standard deviation of the logarithm of the AUC of ridaforolimus is similar to that observed in adults at approximately 0.3, with 10 patients the study would have approximately 96% power to rule out a 25% drop if the true AUC ratio were 1, and approximately 74% power to rule out a 25% drop if the true AUC ratio were 0.9, based on a type I error rate of 0.1 and a one-sided *t* test.

At a safe and well-tolerated dose, ridaforolimus was considered to have obtained sufficient pharmacokinetic properties if the lower bound of the 90% one-sided confidence interval for the geometric mean AUC_0–24h_ on day 5, based on the one-sample *t* test, exceeded 1,304 h•ng/mL, or 75% of the estimated geometric mean AUC_0–24h_ on day 5 after a dose of 40 mg in adults (1,739 h•ng/mL; data on file with ARIAD Pharmaceuticals, Inc./Merck & Co., Inc.).

## SUPPLEMENTARY MATERIALS TABLE


