# Supplementary material for: A phase 1 study of oral ridaforolimus in pediatric patients with advanced solid tumors
Source: Oncotarget. 2016 Oct 4;7(51):84736–47. doi: 10.18632/oncotarget.12450 (PMC5356695; doi:10.18632/oncotarget.12450)
Supplement: Supplementary file 1 [file oncotarget-07-84736-s001.pdf]

A phase 1 study of oral ridaforolimus in pediatric patients with advanced solid tumors

SUPPLEMENTARY TABLE

Supplementary Table S1: Ridaforolimus dosing nomogram in pediatric patients

			Corresponding eligible BSA[a] by ridaforolimus dose (10 mg tablet), m^2				
Dose level[b]	Target dose level, mg/m^2	Actual dose range, mg/m^2 ($\pm$ 2 mg/m^2)	20 mg	30 mg	40 mg	50 mg	60 mg
1	22	24–20	0.83–1.00	1.25–1.50	1.67–2.00		
2	28	30–26	0.67–0.76	1.00–1.15	1.33–1.54	1.67–1.92	
3	33	35–31	0.57–0.65	0.86–0.97	1.14–1.29	1.43–1.61	1.71–1.94

[a]BSA (m^2) = ([height (cm) × weight (kg)]/3,600)$^{1/2}$ as described by Mosteller RD (Simplified calculation of body-surface area. N Engl J Med. 1987; 317:1098). The maximum starting dose was 30 mg in patients aged 6 to 11 years and 40 mg in patients aged 12 to 18 years. For subsequent dose levels, the maximum doses were 40 and 50 mg in patients aged 6 to 11 years, and 50 and 60 mg in patients aged 12 to 18 years.

[b]Two additional planned dose levels (dose level −1, 17 mg/m^2, and dose level −2, 11 mg/m^2) were not used.

BSA, body surface area.
